# Impairment of sleep homeostasis in cervical dystonia patients

**DOI:** 10.1038/s41598-022-10802-y

**Published:** 2022-04-27

**Authors:** Serena Caverzasio, Ninfa Amato, Giacomo Chiaro, Claudio Staedler, Alain Kaelin-Lang, Salvatore Galati

**Affiliations:** 1 Neurocenter of Southern Switzerland, EOC, Via Tesserete 46, 6900 Lugano, Switzerland; 2 Faculty of Biomedical Sciences, Università della Svizzera Italiana (USI), Via Giuseppe Buffi 13, 6900 Lugano, Switzerland; 3 Department of Neurology, Inselspital, Bern University Hospital, Freiburgstrasse 18, 3010 Bern, Switzerland

**Keywords:** Dystonia, Neuroscience

## Abstract

Alterations in brain plasticity seem to play a role in the pathophysiology of cervical dystonia (CD). Since evidences indicate that sleep regulates brain plasticity, we hypothesized that an alteration in sleep homeostatic mechanisms may be involved in the pathogenesis of CD. We explored sleep in control subjects (CTL) and CD patients before (T_pre-BoNT_) and after (T_post-BoNT_) botulinum toxin (BoNT) treatment. A physiological slow wave activity (SWA) power decrease throughout the night was observed in CTL but not in CD at T_pre-BoNT_. BoNT restored the physiological SWA decrease in CD at T_post-BoNT_. Furthermore, in the first part of the night, CD at T_post-BNT_ showed a frontal increase and parietal decrease in SWA power compared to CD at T_pre-BoNT_, with a SWA distribution comparable to that observed in CTL. Our data highlighted a pathophysiological relationship between SWA during sleep and CD and provided novel insight into the transient central plastic effect of BoNT.

## Introduction

Cervical dystonia (CD) is the most prevalent form of focal dystonia^1^, characterized by involuntary contractions of cervical muscles resulting in abnormal postures and repetitive movements of the head and neck^2^.

At present, the gold standard therapy for CD consists of the injection of botulinum toxin (BoNT) in dystonic muscles^2^. Numerous studies have demonstrated the efficacy of this treatment in reducing motor symptoms of CD^3,4^. The injection of BoNT causes the relaxation of the affected muscle by blocking the transmission of acetylcholine at the level of the neuromuscular junction and by influencing ascending somatosensory pathways, within a few days^5,6^. Patients were given regular injections about every 12 weeks since evidence has shown that efficacy begins a few days after the injection and gradually wears off after 9.5–16 weeks^7,8^. Recent data have shown that BoNT, besides its action at the peripheral nervous system, might affect central circuits, by inducing plastic changes in the central nervous system (CNS)^5,9,10^.

Alterations in cortical plasticity seem to play an important role in the pathophysiology of several types of dystonias^11,12^. This is particularly interesting since, in the last years, a growing body of evidence indicates that cortical plasticity is tuned by sleep^13,14^. According to the sleep homeostasis hypothesis, the slow-wave activity (SWA) during non-REM (NREM) sleep is able to downscale the synaptic strength built up during the wake period^13–15^. The SWA acts as a regulator of putative Hebbian plasticity, such as long-term potentiation and depression, preventing saturation of the neuronal network^16^. Learning during wake time directly correlates with the increase of SWA at the beginning of sleep and the following reduction during NREM sleep, as shown in high-density electroencephalography (hdEEG) studies in control subjects (CTL)^15,17^. Furthermore, recent studies suggested that sleep might be implicated in the development and/or modulation of movement disorders, such as Parkinson’s disease (PD)^18,19^. In this regard, a pathophysiological association between SWA-mediated synaptic downscaling disruption and the development of levodopa-induced dyskinesia (LID) in PD patients has been documented^20^.

Although dystonia is mainly a motor disorder, it is important to remark that non-motor symptoms, such as sleep disturbances, are part of the clinical spectrum and negatively affect the quality of life of these patients^21^. To date, only a few studies have addressed sleep in CD patients, showing a reduced sleep quality compared to CTL^22,23^.

In this work, we explored sleep in a group of CD patients before and after the motor benefit of BoNT injection. Furthermore, we investigated the hypothesis that an alteration in sleep homeostasis mechanisms may contribute to the pathogenesis of CD through a whole-night video-polysomnography with high-density electroencephalography (vPSG-hdEEG) study.

## Results

### Demographics features and questionnaires

Demographic and clinical data of all participants are summarized in Table 1. The 8 CD patients have a mean disease duration of 5.1 ± 1.4 (years ± s.e.m), a mean BoNT treatment duration of 3.7 ± 0.7 (years ± s.e.m.) and a mean beneficial effect of BoNT treatment of 7.8 ± 0.4 (VAS scale; mean ± s.e.m.).

**Table 1:** Demographics features and questionnaires.

	CTL, n = 8	CD, n = 8	CTL versus CD (*p*_*MW*_)
**Demographic data**			
Female/male, number	7/1	6/2	ns
Age, year	53.2 ± 4.5	58.6 ± 4.4	ns
**Clinical details**			
Disease duration, year	NA	5.1 ± 1.4	NA
BoNT treatment duration, year	NA	3.7 ± 0.7	NA
Unit of BoNT injected, U	NA	250 ± 29.9	NA
VAS	NA	7.8 ± 0.4	NA
**Questionnaires**			
PSQI	5.6 ± 1.8	5.8 ± 1.3	ns
ESS	7.3 ± 1	6.9 ± 1.5	ns
ISI	4.6 ± 1.7	6.0 ± 3.8	Ns

Mean ± s.e.m; CTL, control subjects; CD, cervical dystonia patients; BoNT, Botulinum toxin injection; VAS, visual analogical scale; PSQI, Pittsburgh sleep quality index; ESS, Epworth sleepiness scale; ISI, Insomnia severity index; yr, years; U, Unit; NA, not applicable; ns, not significant; MW, Mann–Whitney.

The results obtained on the self-administered questionnaires are reported in Table 1. There were no significant differences in sleep quality between CTL and CD (Mann–Whitney, U = 28.5, Z = − 0.372, *p* = 0.710), both presenting pathological values on the PSQI (PSQI > 5), defining them as poor sleepers. Excessive daytime sleepiness, measured through the ESS scale, and insomnia, measured through the ISI scale, were not different between CD and CTL group (ESS, Mann–Whitney, U = 27, Z = − 0.531, *p* = 0.595; ISI, Mann–Whitney, U = 26, Z = − 0.234, *p* = 0.815). Regarding results obtained at the TWSTRS (Table 2), we found a significant decrease of motor symptoms in CD between T_pre-BoNT_ and T_post-BoNT_ (Wilcoxon Signed Ranks Test, Z = − 2.032, *p* = 0.042), as expected.

**Table 2:** Clinical assessment, polysomnographic and actigraphic data in CTL, CD at T_pre-BoNT_ and CD at T_post-BoNT_.

	CTL, n = 8	CD T_pre-BoNT_, n = 5	CD T_post-BoNT_, n = 5	CTL vs CD T_pre-BoNT_ (*p*_*MW*_)	CTL vs CD T_post-BoNT_ (*p*_*MW*_)	CD T_pre-BoNT_ vs CD T_post-BoNT_ (*p*_*WSR*_*)*
**Clinical assessment**						
TWSTRS	NA	12.8 ± 2.3	2.8 ± 0.6	NA	NA	0.042
**Polysomnographic data**						
TST, min	374.4 ± 21	342.8 ± 25.7	362.3 ± 16.6	ns	ns	ns
SL, min	22.6 ± 5.4	25.3 ± 13.3	18.6 ± 10.5	ns	ns	ns
SE, %	83.3 ± 3.5	80 ± 6.1	85 ± 2.2	ns	ns	ns
WASO, min	53.1 ± 3.4	61.2 ± 29.1	43.8 ± 9.3	ns	ns	ns
Wake, %	12.5 ± 3.3	14.8 ± 6.8	10.7 ± 2.2	ns	ns	ns
SWS, %	22.6 ± 1.2	19.6 ± 6.5	20.7 ± 3.7	ns	ns	ns
REM, %	17.1 ± 2.4	16.5 ± 3	19 ± 3	ns	ns	ns
Arousal	16.9 ± 1.6	19.2 ± 3.5	16.4 ± 3.6	ns	ns	0.042

	CTL, n = 5	CD T_pre-BoNT_, n = 3	CD T_post-BoNT_, n = 5	(*p*_*MW*_)	(*p*_*MW*_)	(*p*_*WSR*_*)*
**Actigraphic data**						
eTST, min	413 ± 11.2	394 ± 29.7	378 ± 22.6	ns	ns	ns
eSE, %	85.2 ± 1.1	79.3 ± 4.7	78.1 ± 4.8	ns	ns	Ns

Mean ± s.e.m.; TWSTRS, Toronto Western Spasmodic Torticollis Rating Scale; TST, total sleep time; SL, sleep latency; SE, sleep efficiency; WASO, wakefulness after sleep onset; SWS, slow wave sleep; REM, rapid eye movement; eTST, estimated total sleep time; eSE, estimated sleep efficiency; min, minutes; ns, not significant; NA, not applicable; MW, Mann–Whitney; WSR,Wilcoxon Signed Ranks.

### Polysomnographic and actigraphic data

The descriptive polysomnographic measures of 8 CTL, 5 CD at T_pre-BoNT_ and 5 CD at T_post-BoNT_ are reported in Table 2. No differences were found, either in sleep parameters between CD and CTL, nor between CD at T_pre-BoNT_ and at T_post-BoNT_, other than a significant reduced amount of arousals in the CD group at T_post-BoNT_ (Wilcoxon Signed Ranks Test, Z = − 2.032, *p* = 0.042), similar to the number of arousals observed in CTL. Similarly, no between-group differences were found in the estimated sleep parameters obtained from actigraphic recordings (Table 2).

### Changes in SWA during early and late sleep

**Figure 1:**
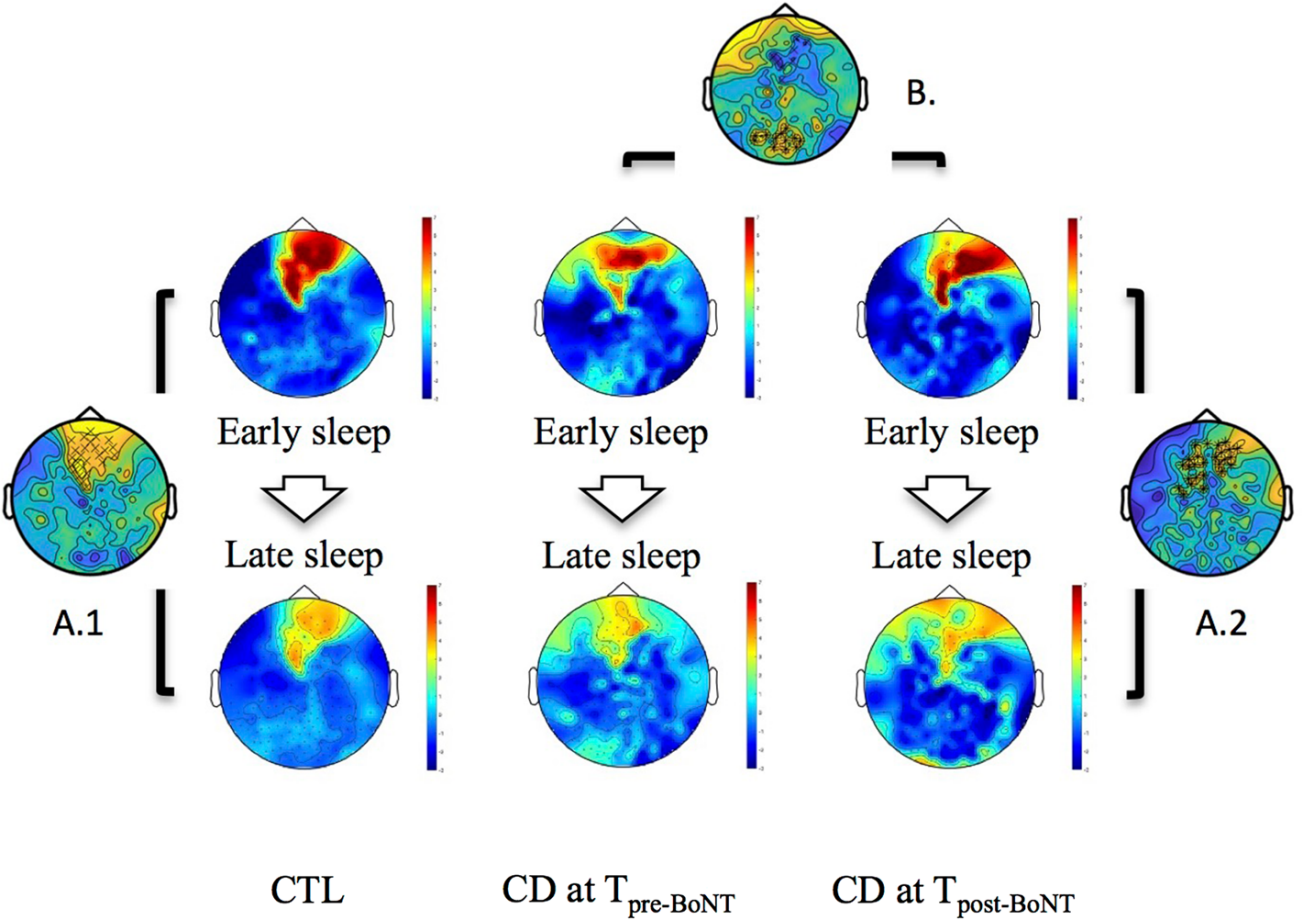
Changes in slow wave activity during early and late sleep. Slow wave activity (SWA) power maps (maximal in red, minimal in blue) during early (upper maps) and late sleep (lower maps) in control subject (CTL, on the left) and in cervical dystonia patients (CD) 1 week before (Time, T_pre-BoNT_, in the middle) and 2 weeks after (Time, T_post-BoNT_, on the right) botulinum toxin injection (BoNT). Statistical maps showing the significant difference between early and late sleep in CTL (A.1; ^x^*p* = 0.041) and in CD at T_post-BNT_ (A.2; *p < 0.001). Statistical map showing the significant difference in the early sleep between CD at T_pre-BoNT_ and CD at T_post-BoNT_ (B; frontal increase, ^x^*p* = 0.042; parietal decrease, *p < 0.001).

Compared between early and late sleep, at T_pre-BoNT_, CD patients presented no differences in SWA power, while they showed a significant overnight SWA power decrease at T_post-BoNT_ (Fig. 1—A.2; *p < 0.001). Similarly, a physiological decrease of SWA power throughout the night was observed in CTL subjects (Fig. 1—A.1; ^x^*p* = 0.041).

In early sleep, a significant difference in SWA was found between CD at T_pre-BoNT_ and at T_post-BNT_. Indeed, SWA power was significantly increased over frontal regions (Fig. 1—B; ^x^*p* = 0.042) and decreased over parietal regions (Fig. 1—B; *p < 0.001) in CD at T_post-BNT._ That was not the case in late sleep, where no differences between CD at T_pre-BoNT_ and at T_post-BoNT_ (ns) were found. Similarly, no differences were observed between CTL and CD at T_pre-BoNT_ (early, ns; late, ns) or CTL and CD at T_post-BoNT_ (early, ns; late, ns) (Fig. 1).

## Discussion

Herein, we highlighted a possible pathophysiological relationship between sleep and CD providing new insights into the effect of BoNT on cortical activity.

We explored subjective and objective characteristics of sleep in a highly selected cohort of patients with CD showing a sustained clinical response to BoNT treatment.

During the selection process, we aimed to exclude all possible confounding variables, in order to investigate the pathophysiological relationship between slow-wave sleep and CD in a highly homogeneous sample, without the misleading impact of other disturbances frequently associated with dystonia (such as depression).

Regarding self-administered questionnaires about sleep quality, we did not find substantial differences between CD and CTL subjects. Previous studies, instead, showed poorer sleep quality in CD patients, measured using the PSQI^23–25^. This inconsistency might be explained by the different exclusion criteria applied and the different group sizes. Indeed, since mood disorders are known to affect sleep perception^23,25^, we excluded depressed patients, whereas CD patients included in previous studies^23,25^, presented mean scores higher than 10 at the BDI scale, which are suggestive of mild depression, according to reference criteria^26^. It is thus possible that sleep disturbances reported in CD are linked to depressive symptoms.

Similarly, we did not find any difference in the ESS between CD and CTL, which is consistent with previous observations^23–25^. However, one study showed that CD patients manifested excessive daytime sleepiness compared to CTL^27^.

In agreement with previous works, we did not find any correlation between disease duration and scores at the subjective sleep questionnaires^24,27^.

As far as objective measures of sleep macrostructure are concerned, we did not find any differences between CD and CTL subjects. Previous vPSG studies on dystonia have shown incongruent results, possibly attributable to high clinical and methodological heterogeneity^22^. Among these studies, to the best of our knowledge, only two focused on CD^23,28^. Lobbezzoo and colleagues found no differences in sleep architecture parameters between CD and CTL^28^, while Antelmi and colleagues reported a reduction of sleep efficiency as well as an increase in sleep latency in CD patients compared to CTL^23^. However, while Lobbezzoo and colleagues performed the vPSG recording in naive patients^28^, Antelmi and colleagues performed it three months after the last BoNT injection^23^. In our work, we did not find any substantial difference in objective sleep parameters between CD and CTL, even though we performed the first vPSG-hdEEG recording before BoNT injection (i.e. 3 months after the last BoNT injection). Nevertheless, we observed a tendency to a decreased sleep efficiency and increased sleep latency in CD patients, which is consistent with the results reported by Antelmi and colleagues^23^.

Our cohort of CD patients underwent vPSG-hdEEG twice, 1 week before and 2 weeks after the scheduled BoNT injection. Of note, we did not observe any change in the sleep macrostructure between the two vPSG-hdEEG recordings, other than a reduced amount of arousals in the vPSG-hdEEG at T_post-BoNT_, despite the clear-cut improvement of motor symptoms after BoNT injection assessed by the TWSTRS.

We extended our analysis to the SWA power and its overnight reduction, reflecting the sleep homeostasis process^13,15,17^. We found that CD patients at T_pre-BoNT_ injection manifested a pathological sustained content of SWA throughout the night. The lack of a substantial overnight decline in this slow activity in CD patients at T_pre-BoNT_, reflecting an impaired synaptic downscaling process, might be linked to the abnormal cortical plasticity extensively described in dystonic patients^11,12,29–31^. Similarly, we have documented elsewhere^20^ that PD patients showing LID had an analogous alteration of the SWA-mediated synaptic downscaling process. This is not surprising, in light of the clinical and neurophysiological features shared by LID and dystonia^32,33^. For instance, part of the clinical spectrum of LID is represented by a dystonic posture of axial muscles^34^. Also, changes in striatal response to cortical stimulation have been reported both in LID and dystonia^35,36^. Above all, an alteration in homeostatic plasticity is a key feature of the pathophysiology of both LID and dystonia^11,12,36,37^.

Remarkably, at BoNT maximal clinical efficacy (T_post-BoNT_), CD patients restored the physiological decrease of SWA throughout the night, associated to a considerable change in the SWA amount in the first part of the night. This corroborates the pathogenic role of sleep homeostasis impairment in the appearance of CD and the hypothesis that BoNT might exert its clinical benefit through CNS-mediated mechanisms^5,9,10^. In this regard, the possible BoNT effect on CNS has been explained either by a retrograde transport or by changes of the afferent sensory input resulting in a reorganization of brain plasticity^5,10,38^. BoNT type A modifies the activity of the spinal recurrent inhibitory pathways, when injected at muscular level, in humans^39^. Moreover, Gilio and colleagues showed that BoNT treatment was able to transiently restore the intracortical inhibition in dystonic patients one month after injection in a transcranial magnetic stimulation study^40^. Likewise, we observed a transient return to a physiological SWA downscaling process after the BoNT injection, comparable to that observed in CTL.

Besides the difference in the SWA decrease during the night, we found differences in its topography as well. The SWA was substantially increased in the frontal region and reduced in the parietal region in CD at T_post-BoNT_, supporting the central effect of BoNT and the consequent cortical reorganization^9^.

Of note, the SWA distribution over the scalp observed in CD patients after the treatment was comparable to that observed in CTL subjects.

Interestingly, our finding of an increased SWA over posterior parietal regions seems to be consistent with a previous observation concerning the execution of the sensory trick^41^. The sensory trick is a specific maneuver usually adopted throughout the day by CD patients to ameliorate dystonia; a sensory stimulus (i.e. touching the cheek) yields a change in dystonic muscles contraction^42^. The authors argued that the sustained dystonic head deviation causes an adaptation in the brain and, when the sensory trick is applied, the new position is experienced as unbalanced, and such unbalance might result in enhanced activation of the multisensory integration areas lying within the posterior parietal cortex^41^.

Consistently, we found an increase of SWA, which is known to be particularly evident in brain regions heavily activated during day^43^, over these parietal areas. This increase disappeared after BoNT. Taken together, all these findings provide novel insight into the transiently central plastic effect of BoNT and the consecutive reorganization of the brain.

In conclusion, we highlighted a pathophysiological relationship between sleep and CD and provided further evidence of a central effect of BoNT. The small size of our sample, although homogeneous and well-characterized, so to avoid all possible confounders, represented a limitation of our study. A larger, confirmatory study is needed to support the association between sleep and the pathophysiology of CD, as well as the role of BoNT as a temporary SWA-enhancing therapy in CD patients.

## Material and methods

### Subjects

**Figure 2:**
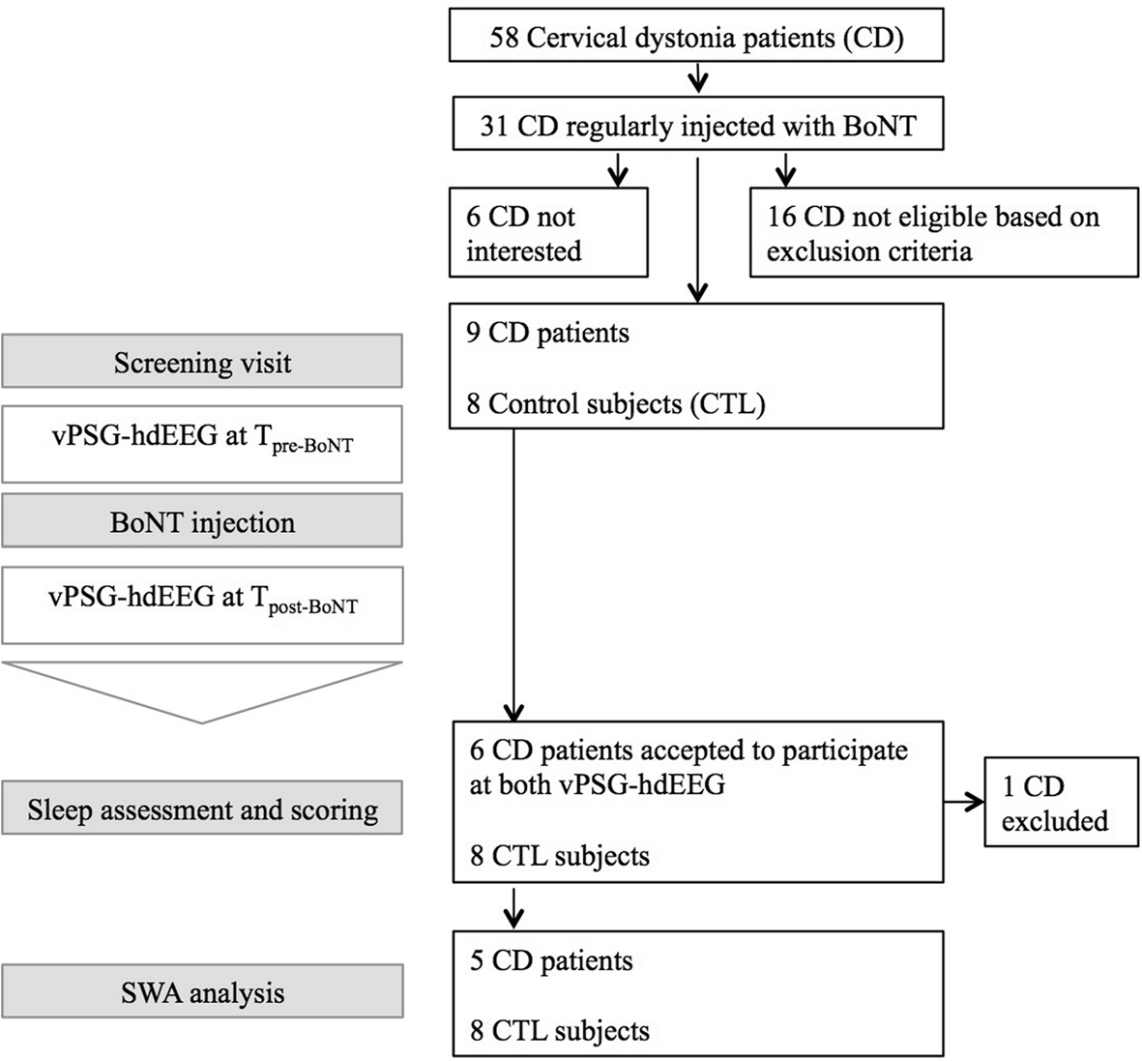
Schematic flow chart of the study and number of subjects enrolled. After the screening visit (Time, T_0_), participants performed two whole-night video polysomnography-high-density electroencephalographic (vPSG-hdEEG) recording, 1 week before (Time, T_pre-BoNT_) and 2 weeks after (Time, T_post-BoNT_) the injection with botulinum toxin (BoNT). The vPSG-hdEEG recordings were assessed and scored. Participants with sleep-related breathing disorders at sleep assessment were excluded from the slow wave activity (SWA) analysis.

Fifty-eight patients with a diagnosis of idiopathic CD fulfilled by movement disorders specialists (S.G. A.K.-L.) according to current criteria^2^, were identified within the database of the Movement Disorders Unit at the Neurocenter of Southern Switzerland. Only patients aged between 30 and 80 years, being submitted to regular (approximately every 12 weeks) injections of BoNT for at least 12 months and showing a sustained beneficial effect, i.e. a decrease of at least 50% on a visual analogical scale (VAS), were considered eligible. Among the 58 CD patients, 31 subjects were under regular treatment with BoNT. Among these, 16 patients were not eligible based on the exclusion criteria listed below, and 6 patients were not interested in participating in the study, thus, 9 patients were included in the present study (see Fig. 2). Eight healthy control subjects (CTL), age and gender-matched, were recruited among colleagues or acquaintances of the researchers. Exclusion criteria for patients and controls included: cognitive impairment (Mini Mental State Examination; MMSE < 24/30); the presence of moderate to severe obstructive sleep apnea disorders (OSAS) and depressive symptomatology (Hospital anxiety and depression scale-Depression section; HADS-D > 10), since the latter has been shown to impact sleep perception^23,25^.

All patients and controls gave their informed consent before participation. The study was approved by the Local Ethics Committee (Comitato Etico Cantonale Ticino) and conducted in compliance with the current version of the Declaration of Helsinki, the ICH-GCP and all national regulatory requirements.

### Experimental design

During the screening visit (2 weeks before BoNT injection; Time, T_0_), all participants were assessed with specific questionnaires relative to their non-motor symptoms, such as mood disorders (Hospital Anxiety and Depression Scale (HADS)), sleep quality (Pittsburgh sleep quality Index (PSQI)), daytime sleepiness (Epworth Sleepiness Scale (ESS)) and insomnia (Insomnia severity index (ISI)). Motor symptoms were assessed using the severity subscale of the Toronto Western Spasmodic Torticollis Rating Scale (TWSTRS). The motor symptoms assessment was repeated 1 week after the BoNT injection.

Six out of 9 CD patients accepted to undergo a vPSG-hdEEG recording 1 week before the BoNT injection (Time, T_pre-BoNT_), i.e. at the nadir of the clinical benefit of the previous injection, and 2 weeks after BoNT treatment, i.e. at the maximum clinical effect of the BoNT (Time, T_post-BoNT_) (Fig. 2). After the sleep assessment, 1 CD patient was excluded from further analysis because of moderate to severe sleep apnea (apnea–hypopnea index (AHI) ≥ 15 per hour), which is known to affect normal sleep architecture^44^. Therefore, 5 CD and 8 CTL were included in the final sleep analysis (Fig. 2).

### Actigraphy

During the week preceding the two vPSG-hdEEG, patients were asked to wear an actigraph on the non-dominant wrist to assess their wake-sleep cycle^45^. The last day of actigraphic recording coincided with the vPSG-hdEEG. Two out of 5 CD at T_pre-BoNT_ and 3 out of 8 CTL did not undergo actigraphic monitoring due to technical failure. The device (Respironics Actiwatch Spectrum Plus, Philips) recorded data continuously using 30-s sampling epochs. Data were scored using a validated algorithm included in the commercial software and the estimated total sleep time (eTST), estimated time in bed (eTIB) and estimated sleep efficiency (eSE) were obtained.

### Whole-night vPSG-hdEEG recording

The whole-night vPSG-hdEEG recording was performed in a dark, sound-attenuated laboratory room at the sleep center. The recording started between 10:30 and 11:30 p.m., based on the participant’s usual bedtime and terminated upon its spontaneous awakening. Polysomnographic recordings included 256 EEG channels (Net Station System 200, v4.0, Electrical Geodesics), bilateral electrooculogram (EOG), submental and anterior tibialis electromyogram (EMG), cardiorespiratory channels, electrocardiogram (ECG), oro-nasal airflow (nasal cannula), microphone, thoracic and abdominal effort (piezoelectric strain gauges), arterial oxygen saturation (pulse oximetry with finger probe) and video.

To reduce interscorer variability, all recordings were scored blind by one accredited clinical polysomnographist (G.C.), using standard criteria of the American Association of Sleep Medicine.

### hdEEG analysis

High-density EEG data were sampled at 250 Hz and offline bandpass Finite Intense Response (FIR) filtered between 0.5 and 40 Hz. Non-rapid eye movement (NREM) sleep data were extracted, rejecting epochs containing arousals, recombined and subdivided in 2 equal parts, which were then considered as early and late sleep respectively and were further analyzed using EEGLAB^46^, Fieldtrip^47^ and custom made MATLAB (The MathWorks, Inc., Natick, Massachusetts) codes. Data were visually inspected to exclude artifacts. An independent component analysis (ICA) was performed, using an improved implementation of the infomax algorithm^48^, available in EEGLAB, and components ascribable to artifacts were excluded. Bad channels were identified, rejected and replaced using spherical interpolation (max. 15% of channels). Time–frequency decomposition was performed on re-referenced (to the average), downsampled (to 128 Hz), four seconds EEG epochs, with a two second overlap. Then, EEG power for each electrode, was normalized to the average of all the electrodes.

### Statistical analysis

First, data were examined for normality, using the Shapiro–Wilk test, and homogeneity of variance, using the Levene test. Since data did not meet the required assumptions to be analyzed by means of parametric methods, the Mann–Whitney U test for independent data and the Wilcoxon-signed rank test for related data were used to compare respectively between and within-subjects. Correlation analyses were performed using the non-parametric Spearman test. Statistical Package for the Social Sciences version 20 (SPSS, Inc., Chicago, IL) was used for the analysis.

Differences within (early vs late sleep) and between (T_pre-BoNT_/T_post-BoNT_/CTL) groups, concerning spectra, were investigated by means of a statistical non-parametric cluster-based permutation test^49,50^. Statistical significance was set at a *p* value < 0.05.

## Data Availability

The datasets are available from the corresponding author on reasonable request.
